# GRN and KLRB1 define a shared peripheral-blood transcriptomic signature linking SLE and IPF

**DOI:** 10.1016/j.jtauto.2026.100357

**Published:** 2026-02-20

**Authors:** Lijun Pang, Yunfei Li, Junjie Chen, Shuangshuang Shang, Ming Li, Chuanbing Huang

**Affiliations:** Department of Rheumatology, The First Affiliated Hospital of Anhui University of Chinese Medicine, Hefei, Anhui, 230038, China

**Keywords:** GRN, Idiopathic pulmonary fibrosis, KLRB1, Parsimonious diagnostic model, Peripheral blood transcriptome, Systemic lupus erythematosus

## Abstract

**Background:**

Systemic lupus erythematosus (SLE) and idiopathic pulmonary fibrosis (IPF) share immune–inflammatory features, yet their convergent peripheral-blood transcriptomic signatures remain incompletely defined. We sought to identify shared blood gene programs linking SLE and IPF, prioritize robust cross-disease markers, and evaluate parsimonious diagnostic models with experimental and external assessments.

**Methods:**

Peripheral-blood transcriptomes were analyzed in GEO discovery cohorts (SLE: GSE49454; IPF: GSE33566). Differential expression (limma) and weighted gene co-expression network analysis (WGCNA) were performed separately per disease, and concordant shared signals were integrated to form a shared candidate pool. Consensus feature selection combined LASSO logistic regression, nested cross-validated SVM–RFE, and random forest to derive parsimonious gene panels for SLE and IPF. Logistic-regression models were trained in discovery cohorts and externally validated in independent cohorts (SLE: GSE65391, GSE72509; IPF: baseline samples from longitudinal GSE93606). Experimental validation was conducted in an independent hospital cohort (60 SLE, 30 healthy controls) using PBMC RT–qPCR and serum GRN ELISA, with correlation and covariate-adjusted association analyses. Fixed models were additionally applied without refitting to non-target inflammatory cohorts (RA: GSE93272; ICU sepsis/non-infectious critical illness: GSE134347).

**Results:**

Discovery analyses identified 389 SLE and 248 IPF DEGs and yielded 43 concordantly regulated shared DEGs; WGCNA identified 43 shared module genes, producing a non-redundant shared candidate pool of 78 genes enriched for B-cell and myeloid programs. Consensus selection generated a 6-gene SLE panel (EIF2AK2, GRN, ASGR2, KLRB1, LGALS9, KLF13) and a 4-gene IPF panel (GRN, ARG1, KLRB1, FCMR). The SLE model achieved AUC 0.996 in discovery and validated at AUC 0.888 (GSE65391) and 0.761 (GSE72509); the IPF model achieved AUC 0.906 in discovery and 0.722 in baseline validation. In the hospital cohort, RT–qPCR confirmed dysregulation of the six-gene panel, and serum GRN was markedly elevated in SLE (median [IQR] 43.58 [38.44–54.42] vs. 14.26 [12.79–15.26] ng/mL). Within SLE, serum GRN correlated with SLEDAI and inversely with C3/C4 and WBC; after covariate adjustment, associations with WBC, ESR, C3, and C4 remained significant, whereas associations with hs-CRP and SLEDAI were attenuated. In non-target cohorts, the SLE model showed moderate discrimination for RA (AUC 0.73) but limited discrimination for ICU sepsis (AUC 0.64) and none for non-infectious critical illness (AUC 0.50), while the IPF model showed minimal discrimination for RA (AUC 0.51) but high discrimination for ICU groups (AUC 0.99 and 0.96).

**Conclusions:**

GRN and KLRB1 anchor a shared peripheral-blood transcriptomic signature linking SLE and IPF, enabling parsimonious diagnostic models with multi-cohort validation and clinical experimental support. External in silico applications to other inflammatory contexts indicate context-dependent model behavior, underscoring the importance of cohort-appropriate interpretation and validation.

## 1 Introduction

Systemic lupus erythematosus (SLE) is a prototypical systemic autoimmune disease characterized by loss of self-tolerance, production of diverse autoantibodies and multisystem organ involvement, typically affecting young women [1]. Despite advances in immunosuppressive therapy and treat-to-target strategies, many patients still experience persistent disease activity, flares and cumulative organ damage [2]. Conventional laboratory markers such as complement levels and acute-phase reactants are imperfect surrogates of disease activity and do not capture the underlying molecular heterogeneity of SLE [3,4]. Whole-blood transcriptomic studies have consistently identified reproducible immune-expression programs, providing a practical window into systemic immune dysregulation and enabling biomarker nomination beyond routine serology [5,6].

Pulmonary involvement in SLE is clinically heterogeneous, and interstitial lung disease (ILD) represents a severe complication associated with significant mortality [[7], [8], [9]]. Recent evidence syntheses and the 2025 ERS/EULAR guidelines emphasize the need for longitudinal monitoring, yet minimally invasive biomarkers remain limited [10,11]. However, public transcriptomic cohorts directly comparing SLE-ILD with SLE without lung involvement are scarce. This data scarcity limits systems-level biomarker discovery in this specific domain. To bridge this gap, we leveraged Idiopathic Pulmonary Fibrosis (IPF) as a prototypical model of fibrosing lung disease. While the initiating etiologies of SLE and IPF are distinct, emerging evidence indicates convergent downstream pathways [12]. These shared mechanisms include aberrant innate immune activation, myeloid cell dysregulation, and cytokine-mediated fibroblast activation [[13], [14], [15]]. Consequently, comparing the transcriptomic landscapes of SLE and IPF offers a unique strategy to disentangle the complex heterogeneity of lupus. By identifying shared molecular signatures, we aim to isolate specific “fibro-inflammatory” gene programs distinct from general autoimmune noise. This approach potentially highlights mechanisms relevant to lung vulnerability in SLE patients.

Peripheral blood transcriptomics provides a scalable readout of circulating immune states and supports biomarker development. In IPF, a validated 52-gene peripheral-blood expression profile stratifies transplant-free survival across independent cohorts [16,17]. In SLE, co-expression network analyses and supervised feature selection have been used to identify reproducible blood modules and compact diagnostic gene panels [18]. Recent whole-blood RNA studies further support transcriptome-based SLE stratification and endotype validation in independent cohorts [19]. However, systems-level comparisons that directly relate SLE blood programs to prototypical fibrosing lung disease remain uncommon in public resources. In addition, many reported signatures are developed within a single cohort or platform and are not rigorously tested for cross-platform robustness or disease specificity against other inflammatory conditions. These gaps limit clinical interpretability and motivate cross-disease, cross-platform, and specificity-aware validation.

In searching for convergent mediators, progranulin and CD161 represent two complementary immune axes. Progranulin (PGRN), encoded by GRN, is a secreted glycoprotein involved in lysosomal homeostasis and tissue repair [20]. PGRN can be cleaved into granulin peptides, and its inflammatory effects vary by context [21]. PGRN has been linked to TNF pathway regulation, but direct binding to TNF receptors remains debated [22,23]. In SLE, circulating PGRN is elevated and correlates with disease activity in clinical cohorts [24,25]. Experimental and review evidence also connects PGRN to inflammatory and fibrotic tissue remodeling [26]. CD161, encoded by KLRB1, is a C-type lectin receptor expressed on natural killer cells and on IL-17-biased T-cell subsets, and it can shape effector responses during chronic inflammation [27]. Altered CD161-positive lymphocyte compartments have been reported in SLE [28]. Together, GRN and KLRB1 provide a biologically plausible myeloid-lymphoid pair to interrogate in peripheral-blood transcriptomes. Because both molecules vary across inflammatory and degenerative states, disease specificity should be tested in appropriate inflammatory-disease controls [29].

In this study, we integrated peripheral-blood transcriptomes from SLE and IPF to map direction-consistent shared signals and construct a non-redundant cross-condition candidate set. We combined differential expression with WGCNA-based module discovery, followed by consensus feature selection to derive parsimonious diagnostic gene panels for SLE and IPF. Model performance was evaluated by fully nested cross-validation in the discovery cohorts and further tested in independent external datasets. We then performed clinical validation in an independent SLE cohort using PBMC RT–qPCR and serum PGRN ELISA, and related PGRN to disease activity and serological indices with covariate adjustment. Finally, to probe disease-context specificity, we applied the fixed models without refitting to non-target inflammatory cohorts, including rheumatoid arthritis and critical illness.

## 2 Materials and methods

### 2.1 Public transcriptomic datasets and preprocessing

Peripheral-blood transcriptome datasets were obtained from the Gene Expression Omnibus (GEO). Three SLE cohorts were included: GSE49454 (whole-blood microarray, 157 SLE and 20 healthy controls), GSE65391 (whole-blood microarray, 924 SLE and 72 healthy controls; the processed, batch-corrected expression matrix provided by the original study/GEO was used directly), and GSE72509 (PAXgene whole-blood RNA-seq, 99 SLE and 18 healthy controls). Two IPF cohorts were included: GSE33566 (whole-blood microarray, 93 IPF and 30 healthy controls) and GSE93606 (external validation cohort). For the longitudinal GSE93606 dataset, we rigorously selected only the baseline samples (Timepoint 0) to ensure independence. Three IPF patients lacked baseline genomic data and were excluded, resulting in a final validation set of 57 IPF patients and 20 healthy controls. These datasets are summarized in Table 1.

**Table 1:** Summary of the GEO datasets involving SLE and IPF patients.

ID	GSE number	Platform	Samples	Source type	Disease	Group
1	GSE49454	GPL10558	157 patients and 20 controls	Whole blood	SLE	Discovery cohort
2	GSE65391	GPL10558	924 patients and 72 controls	Whole blood	SLE	Validation cohort
3	GSE72509	GPL16791	99 patients and 18 controls	PAXgene whole blood RNA-seq	SLE	Validation cohort
4	GSE33566	GPL6480	93 patients and 30 controls	Whole blood	IPF	Discovery cohort
5	GSE93606	GPL11532	57 patients and 20 controls	Whole blood	IPF	Validation cohort

Preprocessing followed dataset-specific processing levels. For GEO microarray cohorts provided as series matrices (GSE49454 and GSE33566), series matrix files and corresponding platform annotation files were downloaded. Expression value distributions were inspected to determine whether log2 transformation was required, and log2(x + 1) transformation was applied when necessary. Probe IDs were mapped to official gene symbols; probes without valid gene symbols were removed, and multiple probes mapping to the same gene were collapsed at the gene level using the median value. For GSE65391, we directly used the batch-corrected expression matrix provided by the original study/GEO without additional normalization or batch correction. For GSE72509, we used the GEO-provided gene-level expression file and applied log2 transformation prior to downstream analyses. To address platform heterogeneity and technical variation during external validation, cohort-specific Z-score standardization was applied prior to diagnostic model evaluation. Standard within-cohort quality control included distribution checks and PCA-based inspection; analyses were conducted within each cohort separately to avoid cross-platform information leakage, and expression matrices were not merged across cohorts.

### 2.2 Identification of differentially expressed genes

Differential expression analysis was performed in the discovery cohorts (GSE49454 for SLE and GSE33566 for IPF) using limma. After probe-to-gene mapping and within-cohort normalization, genes with consistently low signal were removed using microarray-appropriate intensity/variance filtering. A gene-wise linear model was fitted with disease status (case vs control) as the primary predictor, using a design matrix of the form ∼ group (no additional covariates were included because harmonized sample-level clinical covariates were not consistently available across GEO series matrices). Empirical Bayes moderation was applied to improve variance estimation, and P values were adjusted by the Benjamini–Hochberg method. Genes with |log2FC| > 1 and FDR <0.05 were defined as DEGs. Shared DEGs between SLE and IPF were obtained by intersecting disease-specific DEG lists, and only concordantly regulated genes (shared up-regulated and shared down-regulated) were retained for downstream analyses.

### 2.3 Weighted gene co-expression network analysis

WGCNA was conducted separately in the SLE discovery cohort (GSE49454) and the IPF discovery cohort (GSE33566) using normalized gene-level expression matrices to identify disease-associated co-expression modules. Genes were ranked by median absolute deviation (MAD), and the top 8000 most variable genes were retained for network construction. Sample quality was assessed using goodSamplesGenes and hierarchical clustering to identify potential outliers. Soft-thresholding powers were evaluated using pickSoftThreshold (powerVector = 1–10 and 12–20, step = 2), and the power achieving an approximately scale-free topology (signed R^2^ ≈ 0.85) was selected. Networks were constructed using blockwiseModules with an unsigned topological overlap matrix (TOMType = “unsigned”), minimum module size of 30, deepSplit = 2, reassignThreshold = 0, and module merging cut height of 0.25. Module eigengenes (MEs) were correlated with disease status using Pearson correlation to identify disease-associated modules; genes within significant modules were extracted for each disease, and the overlap between SLE- and IPF-derived module genes was defined as shared WGCNA genes.

### 2.4 Protein–protein interaction network and functional enrichment analysis

A non-redundant shared candidate pool (n = 78), defined as the union of concordant shared DEGs and shared WGCNA genes, was used for protein–protein interaction (PPI) and functional enrichment analyses. The candidate genes were uploaded to STRING (*Homo sapiens*) to construct a PPI network using a medium confidence threshold (interaction score ≥0.4), and the resulting network was visualized in Cytoscape; node degree was used to summarize highly connected hub genes. Functional enrichment analyses for Gene Ontology (GO) and Kyoto Encyclopedia of Genes and Genomes (KEGG) pathways were performed using clusterProfiler, with the background universe defined as all genes passing preprocessing and filtering in the corresponding discovery cohort (i.e., the analyzable gene set for GSE49454 or GSE33566, rather than the whole genome), to avoid biased enrichment due to platform-specific gene coverage. Over-representation analysis used hypergeometric testing, and multiple testing was controlled using Benjamini–Hochberg FDR correction; adjusted P < 0.05 was considered statistically significant.

### 2.5 Machine learning–based consensus feature selection and construction of parsimonious diagnostic gene panels

To derive parsimonious diagnostic gene panels for SLE and IPF from the shared candidate pool (n = 78), we implemented a consensus machine-learning framework integrating LASSO logistic regression, nested cross-validated SVM–recursive feature elimination (SVM–RFE), and random forest (RF). All modelling was conducted within each cohort separately and expression matrices were not merged across cohorts to prevent cross-platform information leakage. We separated model development from performance estimation. The final fixed gene panels reported in the manuscript were derived once in the full discovery cohort using the consensus strategy described below and were then used for external validations without refitting. For unbiased internal estimation, we implemented a fully nested cross-validation pipeline. Within each outer fold, feature selection was repeated de novo from the 78-gene candidate pool (LASSO + SVM–RFE + RF → consensus intersection → logistic regression), and out-of-fold predictions were aggregated to obtain the nested-CV ROC/AUC.

For LASSO, binomial models were fitted using glmnet (α = 1). In the SLE discovery cohort, the penalty parameter λ was selected by 10-fold cross-validation using deviance as the optimization criterion (cv.glmnet, type. measure = “deviance”), and λ.1se was adopted to enforce parsimony and reduce overfitting; genes with non-zero coefficients at λ.1se were retained as the LASSO-selected SLE features. In the IPF discovery cohort, λ was likewise determined via 10-fold cross-validation, and λ.min was used to avoid overly sparse selections under cohort heterogeneity and to preserve sensitivity for downstream cross-algorithm overlap; genes with non-zero coefficients at λ.min were retained as the LASSO-selected IPF features.

For SVM–RFE, feature selection was implemented under a 5-fold nested cross-validation design. The outer 5 folds provided an unbiased internal estimate and enabled stability assessment, while SVM–RFE was conducted within each outer training split using caret’s RFE procedure with a radial basis function (RBF) SVM (method = “svmRadial”). Inner-loop optimization targeted discrimination (ROC/AUC) with probabilistic outputs enabled (classProbs = TRUE; summaryFunction = twoClassSummary; metric = “ROC”). Feature-selection stability was quantified by recording selected features across outer folds and summarizing gene-wise selection frequency; genes selected in at least 3 of 5 outer folds were retained as the SVM–RFE consensus set.

For RF, classifiers were trained using randomForest with 500 trees (ntree = 500) and default mtry. Variable-importance scores were computed within each outer training fold and the mean importance was aggregated across outer folds; the top 20 genes by mean importance were retained as the RF importance set. For each disease, cross-algorithm consensus diagnostic feature sets were defined as the overlap among (i) LASSO-selected genes (SLE: λ.1se; IPF: λ.min), (ii) SVM–RFE frequency-based consensus genes (≥3/5 outer folds), and (iii) RF top-20 importance genes. Random seeds were fixed to ensure reproducibility. Key packages included glmnet, caret, e1071, randomForest, pROC, and ggplot2.

### 2.6 Statistical analysis and model evaluation

Diagnostic models for SLE (EIF2AK2, GRN, ASGR2, KLRB1, LGALS9, and KLF13) and IPF (GRN, ARG1, KLRB1, and FCMR) were developed using multivariable logistic regression. To mitigate cross-platform scale differences, gene expression values were standardized within each cohort using gene-wise Z-scores (with log2 transformation when appropriate), and all preprocessing was performed independently within each cohort. Models were trained in the discovery cohort and externally validated in independent cohorts (baseline samples only for the longitudinal IPF cohort). Discrimination was assessed by receiver-operating-characteristic (ROC) curves and the area under the ROC curve (AUC), with AUCs and 95% confidence intervals estimated using the DeLong method (pROC:ci.auc); internal discrimination is reported using the nested cross-validation out-of-fold ROC/AUC derived from the modelling pipeline described above. Calibration of the discovery-cohort models was evaluated using bootstrap resampling (B = 500) to obtain bias-corrected calibration curves (rms:calibrate), and calibration intercept and slope were summarized where relevant. Clinical utility was assessed using decision curve analysis (DCA) and clinical impact curves (CIC). Because case–control sampling inflates prevalence, predicted probabilities were intercept-recalibrated for DCA/CIC under an assumed target prevalence of 0.30 (representing a specialist/high-risk setting), and net benefit as well as the number of high-risk individuals and true positives per 1000 patients were calculated across threshold probabilities of 0.05–0.80. Nomograms were generated based on rms:lrm models. All analyses were conducted in R.

### 2.7 Validation of signature gene expression levels

To verify the differential abundance of the identified diagnostic biomarkers, normalized expression values of the final consensus genes were extracted from the respective discovery datasets. The expression distributions of the SLE signature genes (EIF2AK2, GRN, ASGR2, KLRB1, LGALS9, and KLF13) and the IPF signature genes (GRN, ARG1, KLRB1, and FCMR) were compared between disease and healthy control groups. Given the potential non-normal distribution of gene expression data, the non-parametric Wilcoxon rank-sum test (Mann–Whitney *U* test) was employed to evaluate statistical significance between groups. To rigorously visualize the expression patterns and individual sample variability, boxplots overlaid with jittered data points were generated. All statistical comparisons and visualizations were performed using the ggplot2 and ggpubr packages in R, with a two-sided P-value <0.05 considered statistically significant.

### 2.8 Convergent pathway enrichment analysis and hub gene–stratified functional association

To characterize convergent pathway-level signatures shared by SLE and IPF, we performed pre-ranked KEGG gene set enrichment analysis (GSEA) followed by single-sample pathway scoring. In the SLE (GSE49454) and IPF (GSE33566) discovery cohorts, differential expression between cases and healthy controls was estimated using limma, and all genes were ranked by the case–control log2 fold change (Case − Control). Pre-ranked GSEA was conducted using clusterProfiler:GSEA with KEGG gene sets curated from MSigDB (c2. cp.kegg.Hs.symbols.gmt) and Benjamini–Hochberg correction for multiple testing. Enrichment results were summarized by normalized enrichment score (NES) and adjusted P values, and visualized as dot plots split by enrichment direction (activated vs suppressed). Key convergent pathways of interest were further displayed using enrichment curves (enrichplot:gseaplot2).

To assess whether cross-disease hub genes were associated with pathway activity at the individual-sample level, we computed single-sample gene set enrichment scores using the GSVA framework (ssGSEA) with the same KEGG gene sets. Within each cohort, samples were stratified into high-vs low-expression groups based on the cohort-specific median expression of GRN or KLRB1. Based on the GSEA results, we focused on KEGG_LYSOSOME (for GRN stratification) and KEGG_NATURAL_KILLER_CELL_MEDIATED_CYTOTOXICITY (for KLRB1 stratification). Differences in ssGSEA scores between strata were evaluated using the two-sided Wilcoxon rank-sum test and visualized as boxplots. All analyses were conducted within each cohort separately to avoid cross-platform information leakage.

### 2.9 Immune cell infiltration and functional stratification analysis

Immune-cell composition in peripheral blood was estimated from transcriptome profiles of the SLE (GSE49454) and IPF (GSE33566) discovery cohorts using CIBERSORT-derived deconvolution outputs, yielding relative fractions for 22 immune cell types (LM22). Immune-cell fractions for 22 LM22 leukocyte subsets were obtained from cohort-specific CIBERSORT output tables and aligned to phenotype files by sample identifiers. Cohort phenotype/group files were harmonized into Control and Case groups (0/0.0 as Control; 1/1.0 as Case), and CIBERSORT matrices were matched to phenotype files by sample identifiers with sample intersection to ensure alignment. Cell types with all-zero fractions or zero variance within a cohort were excluded from the corresponding tests. Global immune landscapes were visualized at the sample level using stacked bar plots. Case–control differences in inferred cell fractions were assessed using two-sided Wilcoxon rank-sum tests. For primary inference across the 22 cell types, P values were adjusted within each cohort using the Benjamini–Hochberg procedure (FDR). For visualization, unadjusted P values are shown. Effect sizes were quantified using Cliff’s delta (Case vs Control), with the sign oriented such that positive values indicate higher median fractions in cases. Cross-disease concordant immune alterations were defined as cell types showing consistent effect directions in both cohorts and meeting the significance criterion in at least one cohort after BH correction, and were summarized by a dumbbell plot linking SLE and IPF effect sizes. Associations between hub/candidate gene expression and immune-cell fractions were evaluated using Spearman’s rank correlation with BH adjustment and displayed as correlation heatmaps. For hypothesis-driven functional stratification, case samples within each cohort were dichotomized into high- and low-expression groups using the cohort-specific median of GRN or KLRB1; pre-specified immune subsets were then compared between strata using two-sided Wilcoxon tests (myeloid/neutrophil-related cells for GRN; NK/T-cell–related subsets for KLRB1), reporting unadjusted P values for these targeted comparisons. All analyses and visualizations were conducted in R using dplyr/tidyr, ggplot2/ggpubr, effsize, psych, and pheatmap.

### 2.10 Independent clinical SLE cohort and sample collection

An independent clinical cohort was established at the First Affiliated Hospital of Anhui University of Chinese Medicine to experimentally validate the transcriptome-derived diagnostic signature. SLE patients were classified according to the 2019 EULAR/ACR criteria, and healthy controls (HCs) were age- and sex-matched with no autoimmune, inflammatory, or infectious diseases. Participants were recruited between May 2025 and December 2025 through eligibility screening and voluntary written informed consent (i.e., non-consecutive recruitment based on clinical availability and willingness to participate). Peripheral blood was collected at the time of clinical assessment. PBMCs were prepared for RNA-based assays, and serum was separated and stored at −80 °C until protein quantification. Clinical indices recorded at sampling included SLEDAI, complement C3/C4, hs-CRP, ESR, and white blood cell counts, together with contemporaneous medication exposure (prednisone-equivalent dose, hydroxychloroquine use, and immunosuppressant/biologic exposure). The study was approved by the Ethics Committee of the First Affiliated Hospital of Anhui University of Chinese Medicine (approval No. 2024AH-08) and was conducted in accordance with the Declaration of Helsinki.

### 2.11 PBMC isolation, RNA extraction, and RT–qPCR validation

PBMCs were isolated using a lymphocyte separation medium via density-gradient centrifugation following the manufacturer’s instructions. Total RNA was extracted using a column-based RNA purification kit, and reverse transcription was performed using a commercial first-strand cDNA synthesis SuperMix for qPCR. RT–qPCR was conducted on a real-time PCR system (Bio-Rad CFX Connect) using SYBR Green chemistry. Each 10 μL qPCR reaction contained 5 μL of 2 × SYBR Green master mix, 0.4 μL of forward primer (10 μM), 0.4 μL of reverse primer (10 μM), 0.2 μL of ROX reference dye, 2 μL of cDNA template, and nuclease-free water to volume. Cycling conditions were: 95 °C for 5 min, followed by 40 cycles of 95 °C for 10 s and 60 °C for 30 s; melt-curve analysis was performed to confirm single-product amplification. Relative expression was calculated using the 2ˆ−ΔΔCt method with β-actin as the internal control. The RT–qPCR targets were EIF2AK2, GRN, ASGR2, KLRB1, LGALS9, and KLF13 (primer sequences are provided in the corresponding Supplementary Table S1).

### 2.12 Serum GRN quantification by ELISA

Serum GRN protein levels were measured using a commercial human GRN ELISA kit (MLBio; Cat. No. ml060102) strictly according to the manufacturer’s manual. Briefly, 100 μL of standards or samples were added to pre-coated wells and incubated at 37 °C for 90 min, followed by washing. Biotin-labeled detection antibody (100 μL) was added and incubated at 37 °C for 60 min, washed, and then enzyme conjugate (100 μL) was incubated at 37 °C for 30 min. After washing, substrate solution (90 μL) was added for color development for 15 min protected from light, and the reaction was stopped using stop solution (50 μL). Absorbance was read at 450 nm within 5 min using a microplate reader (RT-6100). Concentrations were derived from a standard curve (4-parameter logistic fit).

### 2.13 In silico specificity assessment in non-SLE/IPF inflammatory cohorts

Independent non-SLE/IPF peripheral-blood transcriptome cohorts were retrieved from GEO for in silico evaluation, including a rheumatoid arthritis (RA) whole-blood cohort (GSE93272) and a critical-illness cohort comprising ICU sepsis and ICU non-infectious critical illness (GSE134347). Dataset identifiers, platforms, specimen sources, and cohort-level group definitions are summarized in Supplementary Table S2.

Within each cohort, expression data were processed independently following the microarray preprocessing principles described above, including inspection of expression distributions and log2 transformation when appropriate, probe-to-gene symbol mapping using platform annotation files, removal of probes without valid symbols, and median-collapsing of multiple probes per gene to obtain a gene-level matrix. Cohort-specific gene-wise Z-score standardization was applied prior to model application. The finalized SLE and IPF logistic-regression models trained in the respective discovery cohorts were then applied to these non-target cohorts without refitting or feature re-selection to generate sample-level model scores and predicted probabilities. Group labels used for evaluation were defined according to GEO phenotype annotations and original study descriptions as summarized in Table S2.

### 2.14 Covariate-adjusted association models for serum GRN

Covariate-adjusted associations between serum GRN and clinical indices were examined using multivariable linear regression with log-transformed serum GRN as the dependent variable. To quantify the independent association of each index, separate models were fitted in which one core predictor (SLEDAI, ESR, WBC, C3, C4, or hs-CRP) was entered per model, while an identical covariate set was retained across analyses. Core predictors were Z-standardized within the SLE cohort; thus, the reported β represents the adjusted change in log(serum GRN) per 1 SD increase in the index, with 95% confidence intervals. The common covariates included age (years), sex, glucocorticoid exposure (prednisone-equivalent dose in mg/day; if unavailable, current glucocorticoid use as a binary proxy), current hydroxychloroquine use, and immunosuppressant/biologic exposure collapsed as Immunosuppressant_any (any current use of MMF/AZA/CYC/biologics). Two alternative approaches were used to account for inflammation/infection-related confounding: Model A additionally adjusted for hs-CRP as a continuous variable and was applied when the core predictor was SLEDAI, ESR, WBC, C3, or C4; Model B replaced hs-CRP with recent infection (within 2 weeks) and, where available, recent antibiotic exposure to avoid conditioning on the exposure of interest when hs-CRP was the core predictor. Model specifications were: Model A, log(Serum GRN) ∼ Core + Age + Sex + Prednisone_equiv_mg_day + Hydroxychloroquine_current + Immunosuppressant_any + hsCRP_mg_L; Model B, log(Serum GRN) ∼ Core + Age + Sex + Prednisone_equiv_mg_day + Hydroxychloroquine_current + Immunosuppressant_any + Recent_infection_2w (+Recent_antibiotics_2w).

### 2.15 Statistical analysis

For experimental validation, PBMC gene-expression levels and serum GRN concentrations were compared between SLE and healthy control groups using two-sided Wilcoxon rank-sum tests, and visualized using boxplots overlaid with individual data points. Associations between serum GRN and clinical indices (SLEDAI, ESR, hs-CRP, C3, C4, and WBC) were evaluated using Spearman’s rank correlation. When multiple correlations were tested simultaneously, P values were adjusted using the Benjamini–Hochberg method. All tests were two-sided, and P < 0.05 was considered statistically significant unless otherwise specified. Analyses were performed in R.

## 3 Results

### 3.1 Differential transcriptomic alterations and shared DEGs between SLE and IPF in peripheral blood

Using the discovery cohorts GSE49454 (SLE) and GSE33566 (IPF), we first assessed global transcriptomic differences between patients and healthy controls. PCA showed an overall separation trend between cases and controls in both datasets (SLE: PC1 = 10.9%, PC2 = 3.5%; IPF: PC1 = 20.1%, PC2 = 11%), indicating disease-associated peripheral-blood transcriptome remodeling (Fig. 1A and B).

**Fig. 1:**
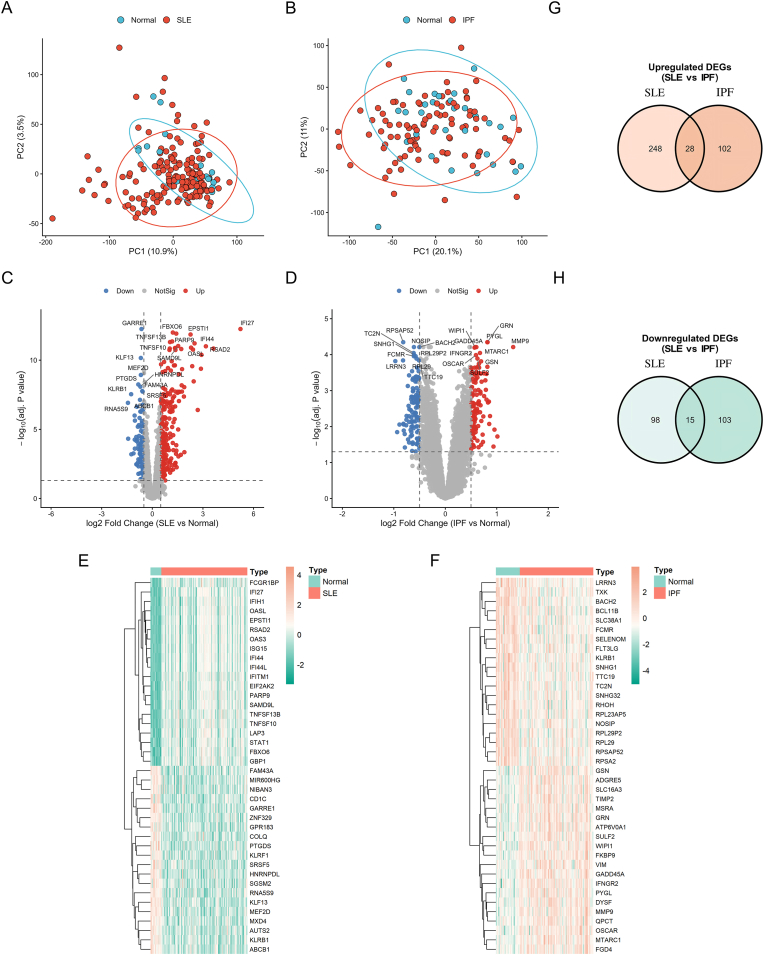
**Global transcriptomic alterations and identification of shared differentially expressed genes (DEGs) in SLE and IPF.** (A, B) Principal Component Analysis (PCA) plots illustrating the global transcriptomic variance and segregation between disease samples (red) and healthy controls (blue) in (A) the SLE discovery cohort (GSE49454) and (B) the IPF discovery cohort (GSE33566). (C, D) Volcano plots displaying the distribution of DEGs identified in (C) SLE and (D) IPF. Red dots indicate significantly upregulated genes, and blue dots indicate significantly downregulated genes (Thresholds: |log2 fold change| > 1 and FDR <0.05). Top DEGs are labeled with gene symbols. (E, F) Hierarchical clustering heatmaps visualizing the expression profiles of the top DEGs, showing distinct separation between patients and controls in (E) SLE and (F) IPF. (G, H) Venn diagrams depicting the intersection of disease-specific DEGs to identify concordant cross-disease signatures. (G) Overlap of upregulated genes showing 28 shared transcripts. (H) Overlap of downregulated genes showing 15 shared transcripts. **Abbreviations:** SLE, Systemic Lupus Erythematosus; IPF, Idiopathic Pulmonary Fibrosis; DEGs, Differentially Expressed Genes; FDR, False Discovery Rate.

Differential expression analysis (|log2FC| > 1 and FDR <0.05) identified 389 DEGs in SLE, including 276 upregulated and 113 downregulated genes (Fig. 1C), and 248 DEGs in IPF, including 130 upregulated and 118 downregulated genes (Fig. 1D). Heatmaps of the top DEGs revealed coherent expression patterns that distinguished patients from controls in each cohort (Fig. 1E and F). Intersection of the SLE and IPF DEG lists yielded 43 concordant shared DEGs, comprising 28 commonly upregulated and 15 commonly downregulated genes (Fig. 1G and H), suggesting overlapping inflammatory and immune-related transcriptional perturbations across SLE and fibrosing IPF.

### 3.2 WGCNA identifies disease-associated co-expression modules shared by SLE and IPF

To characterize coordinated transcriptional programs in peripheral blood, WGCNA was performed in the SLE discovery cohort (GSE49454) and the IPF discovery cohort (GSE33566). Hierarchical clustering of samples did not reveal apparent outliers in either dataset (Fig. 2A–C). Soft-thresholding analysis was conducted to approximate scale-free topology, and the optimal powers were set to β = 12 for SLE and β = 6 for IPF based on scale-independence and mean connectivity profiles (Fig. 2B–D).

**Fig. 2:**
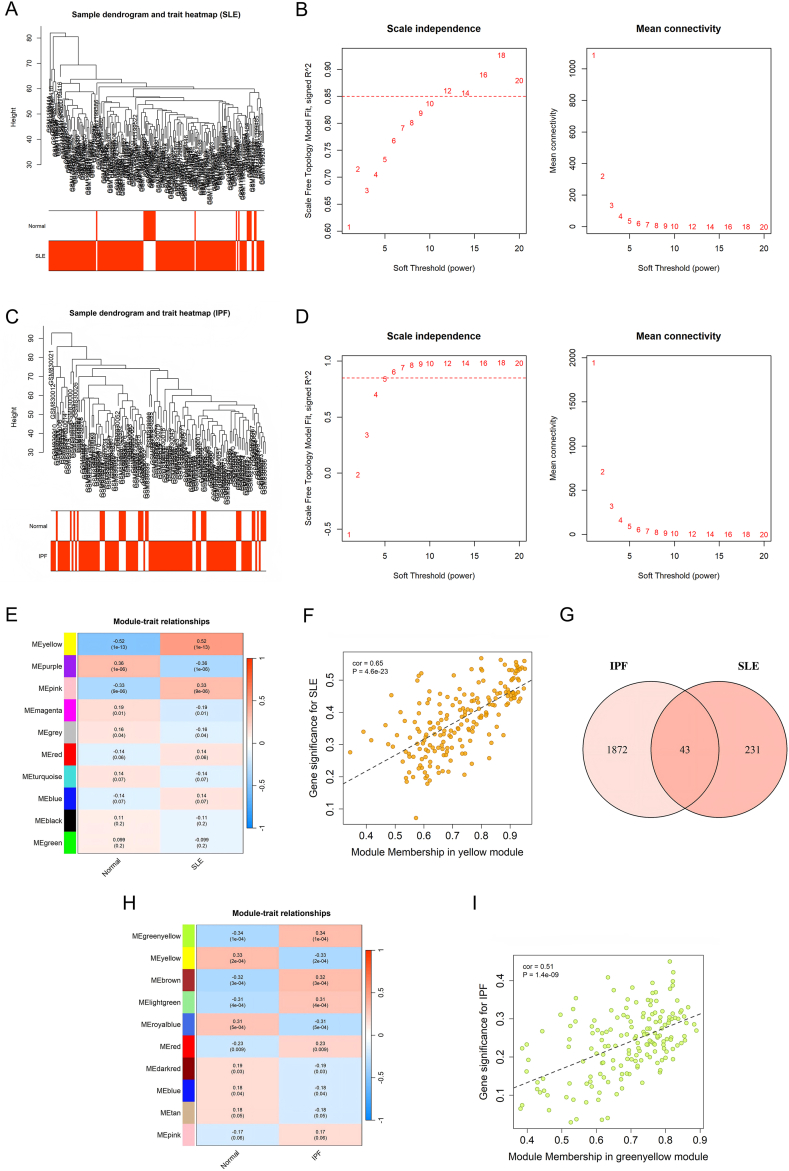
**Weighted Gene Co-expression Network Analysis (WGCNA) identifies shared disease-associated modules in SLE and IPF.** (A, C) Sample dendrograms and trait heatmaps for the SLE discovery cohort (GSE49454) (A) and the IPF discovery cohort (GSE33566) (C), showing no apparent outliers. (B, D) Analysis of network topology for various soft-thresholding powers in SLE (B) and IPF (D). The left panels display the scale-free fit index (signed R^2^), and the right panels show the mean connectivity. The optimal soft-thresholding powers were set at *β* = 12 for SLE and *β* = 6 for IPF. (E, H) Heatmaps of module–trait relationships in SLE (E) and IPF (H). Each cell contains the Pearson correlation coefficient and the corresponding P-value. Red indicates a positive correlation, while blue indicates a negative correlation. Key disease-associated modules (e.g., the yellow module in SLE and the greenyellow module in IPF) show strong correlations with disease status. (F, I) Scatter plots of Gene Significance (GS) versus Module Membership (MM) for the most significant disease-associated modules: the yellow module in SLE (F) (cor = 0.65, P = 4.6 × 10^−23^) and the greenyellow module in IPF (I) (cor = 0.51, P = 1.4 × 10^−9^). (G) Venn diagram displaying the intersection of genes from the identified disease-associated modules in SLE and IPF, revealing 43 shared WGCNA genes. **Abbreviations:** SLE, Systemic Lupus Erythematosus; IPF, Idiopathic Pulmonary Fibrosis; GS, Gene Significance; MM, Module Membership.

Genes were clustered into co-expression modules using dynamic tree cutting with module merging. In the SLE network, module–trait analysis of the top modules indicated that MEyellow was most strongly positively correlated with SLE status (r = 0.52, P = 1 × 10^−13^), MEpink also showed a positive association (r = 0.33, P = 9 × 10^−6^), whereas MEpurple was inversely correlated with SLE (r = −0.36, P = 1 × 10^−6^) (Fig. 2E). In the IPF network, MEgreenyellow showed the highest positive correlation with IPF (r = 0.34, P = 1 × 10^−4^), with additional significant positive associations observed for MEbrown (r = 0.32, P = 3 × 10^−4^) and MElightgreen (r = 0.31, P = 4 × 10^−4^), while MEyellow (r = −0.33, P = 2 × 10^−4^) and MEroyalblue (r = −0.31, P = 5 × 10^−4^) were negatively associated with IPF status (Fig. 2H). The grey category represents genes not assigned to any co-expression module.

To ensure that selected modules were not only correlated with disease status but also enriched for disease-relevant intramodular genes, we further evaluated the relationship between module membership (MM) and gene significance (GS). A strong MM–GS correlation was observed for the SLE yellow module (cor = 0.65, P = 4.6 × 10^−23^) (Fig. 2F), and a comparable association was found for the IPF greenyellow module (cor = 0.51, P = 1.4 × 10^−9^) (Fig. 2I), supporting the biological relevance of these disease-linked modules. Taken together, based on both module–trait correlations and MM–GS relationships, we defined yellow, pink, and purple modules in SLE and greenyellow, yellow, brown, lightgreen, and royalblue modules in IPF as disease-associated modules. Genes within these disease-associated modules were first pooled within each disease, and the resulting SLE and IPF module gene sets were then intersected, yielding 43 shared WGCNA genes (Fig. 2G) that represent cross-disease co-expression signals for subsequent analyses.

### 3.3 Integrated shared DEGs and WGCNA genes define a cross-disease immune interactome enriched for B-cell and myeloid programs

**Fig. 3:**
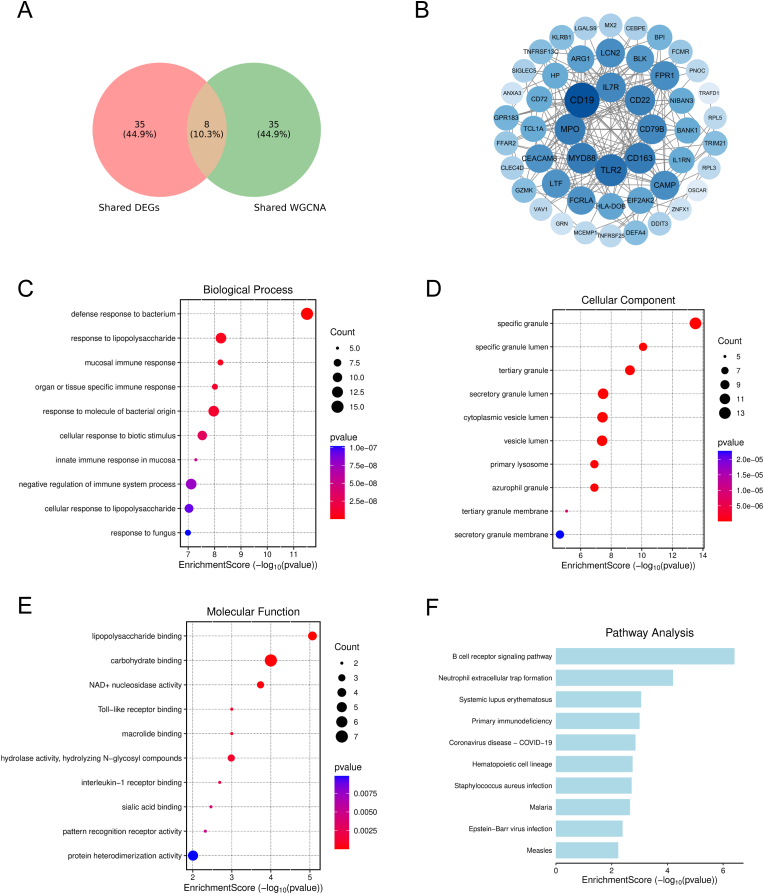
**Integration of shared DEGs and WGCNA genes reveals a cross-disease immune interactome enriched for B-cell and myeloid programs.** (A) Venn diagram illustrating the intersection between concordant shared DEGs (pink) and shared WGCNA genes (green). This integration defined a candidate pool of 78 genes, with 8 core genes (BLK, BPI, CD19, CD79B, GRN, LCN2, LINC00926, and NTNG2) identified by both approaches. (B) Protein–protein interaction (PPI) network of the 78 shared candidate genes constructed via STRING. Node size and color intensity reflect connectivity (degree), highlighting CD19 as a prominent hub and revealing tightly coupled clusters related to B-cell signaling and innate immunity. (C–E) Dot plots summarizing Gene Ontology (GO) enrichment analysis for (C) Biological Process, (D) Cellular Component, and (E) Molecular Function. The size of the dots represents the gene count, and the color gradient indicates significance (–log_10_ P-value). (F) Bar plot displaying the top enriched Kyoto Encyclopedia of Genes and Genomes (KEGG) pathways, ranked by enrichment score. **Abbreviations:** DEGs, Differentially Expressed Genes; WGCNA, Weighted Gene Co-expression Network Analysis; GO, Gene Ontology; KEGG, Kyoto Encyclopedia of Genes and Genomes.

To integrate differential-expression and co-expression evidence across SLE and IPF, we intersected the 43 concordant shared DEGs (Section 3.1) with the 43 shared WGCNA genes derived from disease-associated modules in both cohorts (Section 3.2). Merging these two gene sets generated a non-redundant shared candidate pool of 78 genes, including 8 core genes supported by both approaches—BLK, BPI, CD19, CD79B, GRN, LCN2, LINC00926, and NTNG2 (Fig. 3A). This integrated set was used for subsequent network and enrichment analyses.

We next constructed a STRING-based protein–protein interaction (PPI) network for the 78 shared candidates. The network revealed a densely connected immune interactome, with CD19 emerging as the most prominent hub and additional high-connectivity nodes including TLR2, MYD88, MPO, IL7R, CD79B, CD22, CD163, BLK, and LCN2 (Fig. 3B). The topology highlighted two tightly coupled immune axes: a B-cell–associated module (CD19/CD79B/BLK/CD22/BANK1/TCL1A) and an innate immune/myeloid inflammatory module (TLR2/MYD88/MPO/LCN2/CD163/IL1RN), suggesting coordinated activation of adaptive and innate immune programs shared between SLE and IPF.

Consistent with the network structure, functional enrichment analyses showed strong immune signatures. GO Biological Process terms were enriched for defense response to bacterium, response to lipopolysaccharide, and related biotic stimulus–responsive processes (Fig. 3C). GO Cellular Component enrichment mapped predominantly to specific/azurophil/tertiary granules and secretory granule lumen, supporting a prominent contribution of neutrophil granule biology and degranulation-associated compartments (Fig. 3D). GO Molecular Function terms included lipopolysaccharide binding, Toll-like receptor binding, and other immune receptor–ligand interaction functions (Fig. 3E). KEGG pathway analysis further identified enrichment in B cell receptor signaling pathway, neutrophil extracellular trap formation, and immune-related pathways such as SLE, primary immunodeficiency, and hematopoietic cell lineage (Fig. 3F). Collectively, these results indicate that the shared SLE–IPF transcriptional candidates form an immune-centered interaction network dominated by B-cell signaling and myeloid/neutrophil effector programs, providing a mechanistic basis for downstream biomarker prioritization and model development.

### 3.4 Machine-learning consensus feature selection identifies parsimonious diagnostic gene panels for SLE and IPF

**Fig. 4:**
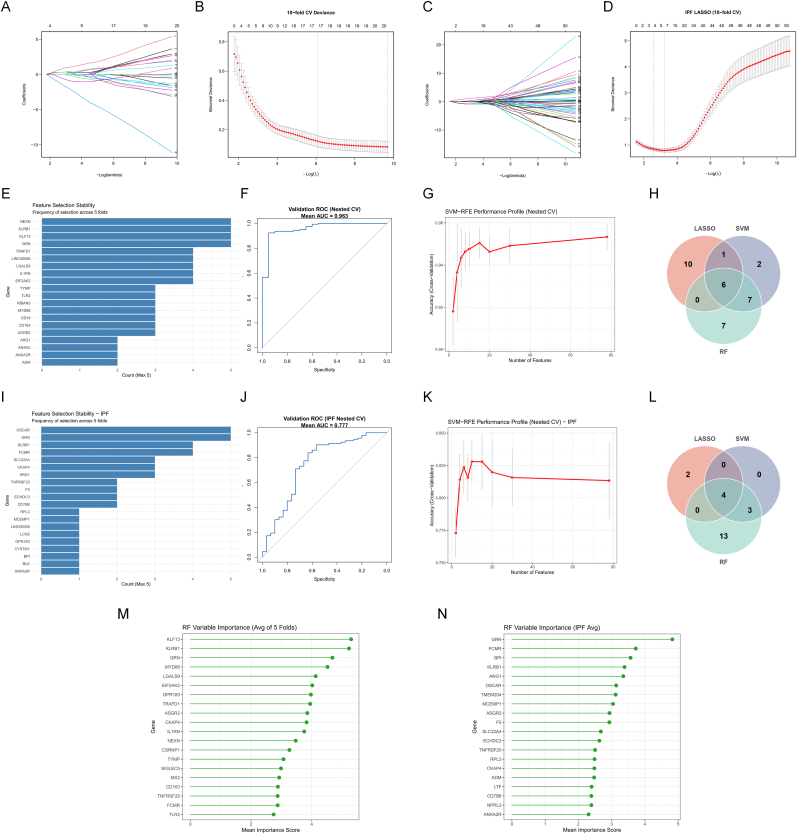
**Identification of parsimonious diagnostic gene panels for SLE and IPF using a machine-learning consensus strategy.** (A–D) Feature selection via LASSO logistic regression. (A, C) Coefficient profiles of candidate genes for SLE (A) and IPF (C). (B, D) Selection of the optimal penalization parameter (λ) using 10-fold cross-validation based on binomial deviance. Dotted vertical lines indicate the minimum deviance (λ_*min*_) and the 1-standard error criterion (λ_*1se*_). λ_*1se*_ was adopted for SLE to enforce parsimony, while λ_*min*_ was used for IPF to preserve sensitivity. (E, G, I, K) Feature selection using nested cross-validated SVM–RFE. (E, I) Bar plots displaying feature selection stability (frequency across 5 outer folds) for SLE (E) and IPF (I). (G, K) Performance profiles showing classification accuracy (y-axis) versus the number of features (x-axis) for SLE (G) and IPF (K). Error bars represent standard deviations. (M, N) Lollipop charts ranking the top 20 genes by Mean Variable Importance derived from Random Forest (RF) models for SLE (M) and IPF (N). (H, L) Venn diagrams illustrating the consensus diagnostic panels. The final gene sets were defined by the intersection of LASSO-selected genes, SVM–RFE stable features (frequency ≥3/5), and top 20 RF importance genes for SLE (H) (6 genes) and IPF (L) (4 genes). (F, J) Receiver Operating Characteristic (ROC) curves evaluating the discrimination of the consensus models based on pooled out-of-fold predictions from nested cross-validation. (F) SLE model (AUC = 0.963). (J) IPF model (AUC = 0.777). **Abbreviations:** LASSO, Least Absolute Shrinkage and Selection Operator; SVM–RFE, Support Vector Machine–Recursive Feature Elimination; RF, Random Forest; AUC, Area Under the Curve.

Using the shared candidate pool (n = 78) derived from concordantly regulated shared DEGs and shared WGCNA module genes, we applied three complementary machine-learning strategies (LASSO, nested cross-validated SVM–RFE, and random forest) to prioritize robust diagnostic features for SLE and IPF (Fig. 4). LASSO logistic regression showed progressive coefficient shrinkage across penalization strengths (Fig. 4A and C) and clear cross-validation optima based on binomial deviance (Fig. 4B and D). To mitigate overfitting and enforce parsimony in the SLE discovery cohort, we adopted the 1-SE rule (λ.1se), yielding a compact LASSO-selected feature set (17 genes). In contrast, for IPF we used λ.min to avoid overly sparse selections under cohort heterogeneity and to preserve sensitivity for downstream cross-algorithm overlap, resulting in six non-zero LASSO-selected features (see Fig. 4A–D).

To obtain an unbiased internal estimate of discrimination and quantify selection robustness, we implemented a 5-fold nested cross-validation SVM–RFE framework with an RBF kernel. The averaged performance profiles across outer folds summarized the relationship between retained feature number and cross-validated performance (Fig. 4F and J). Feature-selection stability was quantified by counting how frequently each gene was selected across the five outer folds; genes selected in ≥3/5 folds were defined as stable features, and the top selection-frequency distributions were visualized for SLE and IPF, respectively (Fig. 4E and I). In parallel, random forest modelling (ntree = 500; default mtry) provided an ensemble-based ranking of discriminative genes; the top 20 genes by mean variable importance were summarized for SLE and IPF (Fig. 4G and K).

Integrating these complementary algorithms, we defined parsimonious consensus panels by intersecting LASSO-selected genes (SLE: λ.1se; IPF: λ.min), SVM–RFE stable features (frequency ≥3/5), and the RF top-20 importance set (Fig. 4H and L). This consensus approach yielded a 6-gene diagnostic panel for SLE (EIF2AK2, GRN, ASGR2, KLRB1, LGALS9, KLF13) and a 4-gene diagnostic panel for IPF (GRN, ARG1, KLRB1, FCMR). Notably, GRN and KLRB1 were shared between the final SLE and IPF consensus panels and were consistently prioritized across algorithms, supporting their robustness as cross-disease blood transcriptomic markers within the SLE–IPF comparative framework. Finally, pooled out-of-fold predictions from the nested cross-validation procedure were aggregated to generate ROC curves (Fig. 4M and N), demonstrating robust discrimination under honest internal estimation (Mean AUC = 0.963 for SLE and 0.777 for IPF).

### 3.5 Construction and evaluation of parsimonious diagnostic models based on consensus gene panels

**Fig. 5:**
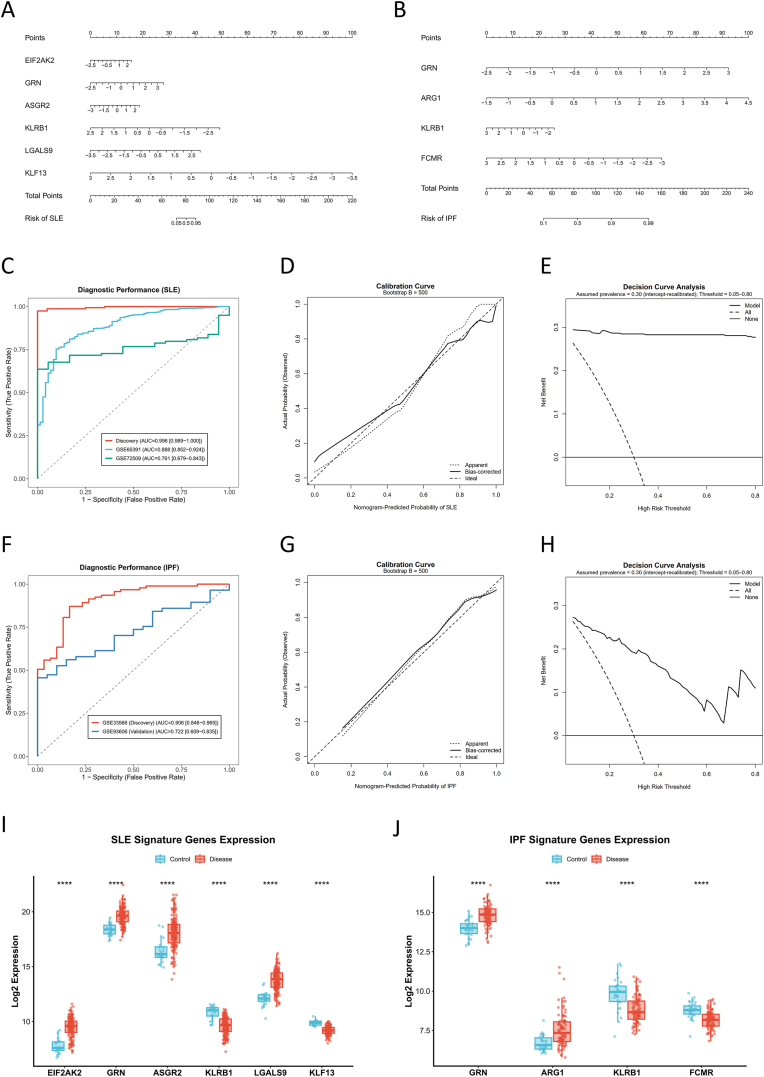
**Construction and evaluation of parsimonious diagnostic models for SLE and IPF based on consensus gene panels.** (A, B) Nomograms developed for predicting the risk of (A) SLE (integrating EIF2AK2, GRN, ASGR2, KLRB1, LGALS9, and KLF13) and (B) IPF (integrating GRN, ARG1, KLRB1, and FCMR). For each patient, gene expression levels are mapped to points on the scale; the total points correspond to the predicted probability of disease. (C, F) Receiver Operating Characteristic (ROC) curves evaluating diagnostic discrimination in the discovery and external validation cohorts for (C) SLE (Discovery: AUC = 0.996; Validation: GSE65391 AUC = 0.888, GSE72509 AUC = 0.761) and (F) IPF (Discovery: AUC = 0.906; Validation: GSE93606 AUC = 0.722). (D, G) Calibration curves assessing the agreement between nomogram-predicted probabilities and observed outcomes for (D) SLE and (G) IPF (Bootstrap B = 500). The diagonal dashed line represents ideal calibration; the solid line represents the bias-corrected performance. (E, H) Decision Curve Analysis (DCA) estimating the clinical net benefit of the diagnostic models for (E) SLE and (H) IPF across a range of threshold probabilities. Analyses were intercept-recalibrated to an assumed target prevalence of 0.30. (I, J) Boxplots validating the differential expression levels of the final signature genes in the discovery cohorts for (I) SLE and (J) IPF. Center lines represent medians; box limits indicate the 25th and 75th percentiles. Statistical significance was determined using the two-sided Wilcoxon rank-sum test (∗∗∗∗*P* < 0.0001). **Abbreviations**: SLE, Systemic Lupus Erythematosus; IPF, Idiopathic Pulmonary Fibrosis; AUC, Area Under the Curve.

Using the cross-algorithm consensus gene panels, we constructed parsimonious logistic-regression diagnostic models for SLE (EIF2AK2, GRN, ASGR2, KLRB1, LGALS9, and KLF13) and IPF (GRN, ARG1, KLRB1, and FCMR). To mitigate cross-platform scale effects, expression values were standardized within each cohort (gene-wise Z-scores, with log2 transformation when appropriate), and all preprocessing and modelling steps were performed independently within each cohort (Fig. 5A and B).

For SLE, the six-gene model achieved excellent discrimination in the discovery cohort (AUC = 0.996, 95% CI 0.989–1.000) and remained performant in external validation cohorts, albeit with expected attenuation under between-cohort heterogeneity (GSE65391: AUC = 0.888, 95% CI 0.852–0.924; GSE72509: AUC = 0.761, 95% CI 0.679–0.843) (Fig. 5C). Model calibration assessed by bootstrap resampling (B = 500) showed overall agreement between predicted and observed probabilities, with acceptable calibration across most of the risk spectrum (Fig. 5D). Decision curve analysis, after intercept recalibration to an assumed prevalence of 0.30 to account for case–control sampling, demonstrated positive net benefit of the model over “treat-all” and “treat-none” strategies across a broad range of threshold probabilities (0.05–0.80), supporting potential clinical utility in specialist or other high-risk settings (Fig. 5E).

For IPF, the four-gene model showed good discrimination in the discovery cohort (AUC = 0.906, 95% CI 0.846–0.965) and moderate performance in the independent validation cohort restricted to baseline samples (AUC = 0.722, 95% CI 0.609–0.835) (Fig. 5F). Bootstrap calibration (B = 500) indicated reasonable concordance between predicted and observed risks (Fig. 5G). In decision curve analysis under the same assumed prevalence (0.30), the model yielded a positive net benefit primarily within low-to-intermediate threshold probabilities, whereas net benefit declined at higher thresholds, suggesting that the model may be most informative for risk stratification when conservative decision thresholds are applied (Fig. 5H).

Finally, differential expression analyses in the respective discovery datasets supported the directional consistency of the finalized panels. In SLE, EIF2AK2, GRN, ASGR2, and LGALS9 were upregulated in cases, whereas KLRB1 and KLF13 were downregulated. In IPF, GRN and ARG1 were upregulated, while KLRB1 and FCMR were downregulated. All genes showed statistically significant case–control differences by the Wilcoxon rank-sum test (two-sided P < 0.05) (Fig. 5I and J).

### 3.6 Pathway concordance analysis based on GSEA and hub gene–stratified GSVA suggests shared immune–lysosomal transcriptional programs between SLE and IPF

**Fig. 6:**
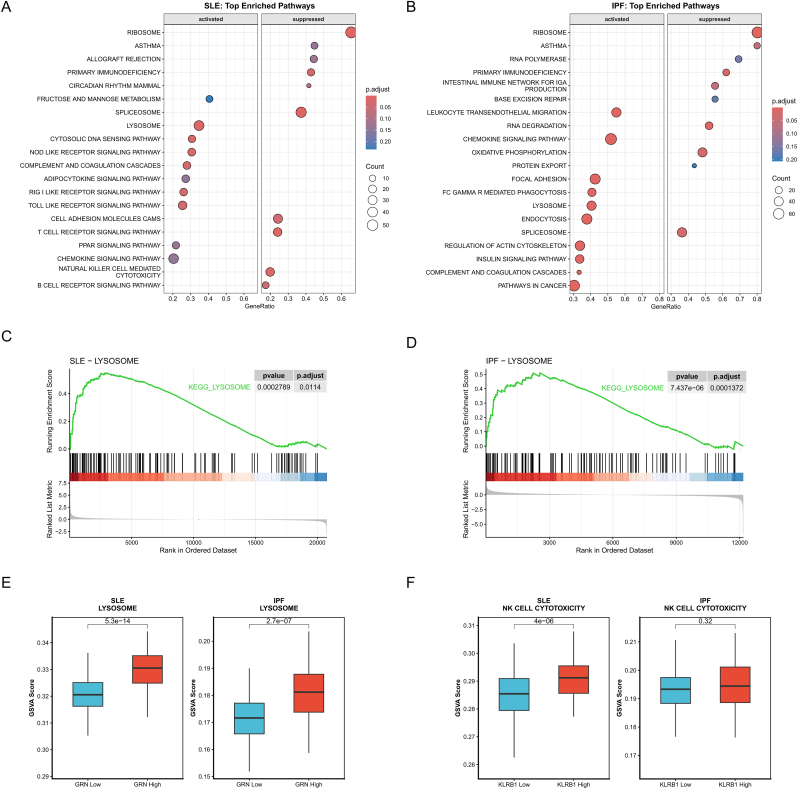
**Pathway concordance analysis based on GSEA and hub gene–stratified GSVA reveals shared immune–lysosomal programs in SLE and IPF.** (A, B) Dot plots summarizing the top enriched KEGG pathways identified by Gene Set Enrichment Analysis (GSEA) in the discovery cohorts for (A) SLE and (B) IPF. Pathways are stratified by activation status, with dot size representing gene count and color indicating adjusted *P*-value. (C, D) GSEA enrichment plots for the “KEGG_LYSOSOME” pathway, demonstrating a concordant positive enrichment pattern in both (C) SLE and (D) IPF. (E) Boxplots validating the association between *GRN* expression and lysosomal pathway activity using Gene Set Variation Analysis (GSVA). Samples were stratified into *GRN*-high and *GRN*-low groups based on cohort-specific median expression. Significantly higher lysosomal scores were observed in the *GRN*-high group for both SLE (left panel; *P* = 5.3 × 10^−14^) and IPF (right panel; *P* = 2.7 × 10^−7^). (F) Boxplots evaluating the association between *KLRB1* expression and the “Natural killer cell mediated cytotoxicity” pathway. In SLE (left panel), the *KLRB1*-high subgroup shows significantly elevated pathway activity (*P* = 4 × 10^−6^). In IPF (right panel), a directionally consistent trend is observed but does not reach statistical significance (*P* = 0.32). Statistical comparisons were performed using the Wilcoxon rank-sum test. **Abbreviations:** GSEA, Gene Set Enrichment Analysis; GSVA, Gene Set Variation Analysis; KEGG, Kyoto Encyclopedia of Genes and Genomes.

To evaluate the biological interpretability of the cross-disease candidate genes at the pathway level, we performed KEGG-based gene set enrichment analysis (GSEA) on the whole-transcriptome profiles of the discovery cohorts for SLE (GSE49454) and IPF (GSE33566), respectively. Both diseases exhibited enrichment signals centered on innate immunity and intracellular degradative systems. In the SLE cohort, LYSOSOME and multiple immune/inflammation-related pathways were among the significantly enriched terms (Fig. 6A). In the IPF cohort, LYSOSOME was likewise significantly enriched, accompanied by enrichment of pathways related to immune-cell migration, phagocytosis/endocytosis, and inflammatory signaling (Fig. 6B). Enrichment-curve visualization further demonstrated a concordant positive enrichment pattern of KEGG_LYSOSOME in both discovery cohorts (Fig. 6C and D), indicating that lysosome-associated transcriptional activation may represent a shared functional phenotype in peripheral blood across SLE and IPF.

Building on these findings, we conducted a “gene-expression stratification–pathway activity validation” for the two consensus cross-disease hub genes (*GRN* and *KLRB1*). Within each cohort, samples were dichotomized into high- and low-expression groups using the cohort-specific median expression of the corresponding gene, and single-sample pathway activity scores were computed using ssGSEA/GSVA. Consistent with the pathway-level enrichment results, the GRN-high subgroup showed significantly higher LYSOSOME pathway activity in both SLE (*P* = 5.3 × 10^−14^) and IPF (*P* = 2.7 × 10^−7^), confirming a robust association across diseases (Fig. 6E). By contrast, KLRB1 stratification was primarily linked to immune effector pathways: in SLE, the KLRB1-high subgroup displayed higher activity of the Natural killer cell mediated cytotoxicity pathway (*P* = 4 × 10^−6^); a directionally consistent trend was observed in IPF, although the association did not reach statistical significance (*P* = 0.32) (Fig. 6F), implying that this relationship may be less stable in the IPF cohort due to heterogeneity or sample size limitations.

Taken together, the concordant enrichment observed by GSEA across discovery cohorts and the hub gene–stratified GSVA results jointly indicate that the shared transcriptomic signal identified in this study extends beyond single-gene differences and is reflected at the pathway level by enhanced lysosome-associated programs along with alterations in selected immune effector pathways (Fig. 6A–F). These findings provide pathway-level evidence supporting the functional relevance of *GRN* and *KLRB1* as cross-disease candidate markers within the SLE–IPF comparative framework.

### 3.7 Immune infiltration landscape and hub-gene–linked functional stratification

**Fig. 7:**
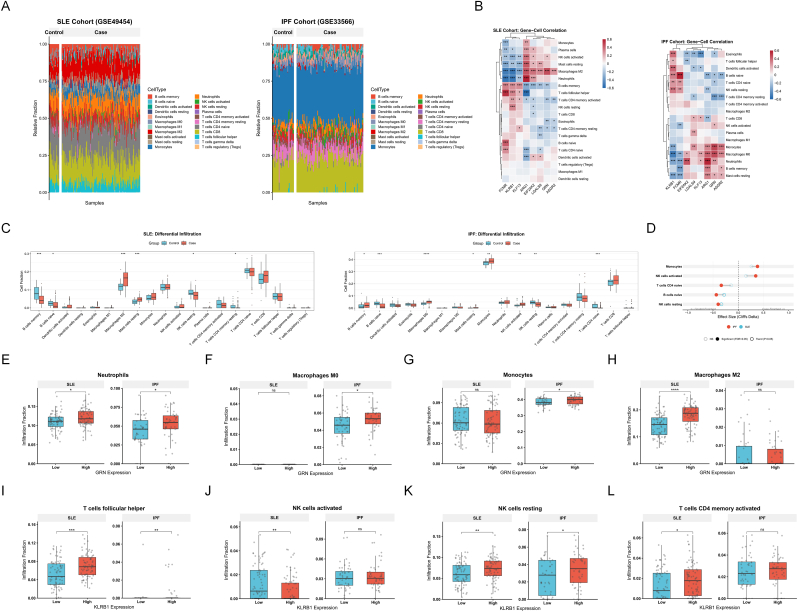
**Immune infiltration landscape and functional stratification analysis linking hub gene expression to specific immune cell alterations in SLE and IPF.** (A) Stacked bar plots visualizing the global composition of 22 immune cell types estimated by CIBERSORT in the SLE (GSE49454) and IPF (GSE33566) cohorts. Each bar represents an individual sample. (B) Heatmaps illustrating Spearman’s rank correlations between the expression of candidate diagnostic genes and infiltrating immune cell fractions. Red indicates positive correlation; blue indicates negative correlation. Significance is denoted by asterisks (∗*P* < 0.05, ∗∗*P* < 0.01, ∗∗∗*P* < 0.001). (C) Boxplots comparing the relative abundance of 22 immune cell subsets between healthy controls (blue) and disease cases (red) in SLE (left panel) and IPF (right panel). (D) Dumbbell plot summarizing cross-disease concordant immune alterations based on effect size (Cliff’s delta). Circles represent the effect size in SLE (blue) and IPF (red); positive values indicate enrichment in cases, while negative values indicate depletion. (E–H) Functional stratification based on *GRN* expression. Case samples within each cohort were dichotomized into *GRN*-low and *GRN*-high groups. Boxplots show differences in the fractions of (E) Neutrophils (elevated in *GRN*-high for both diseases), (F) Macrophages M0, (G) Monocytes, and (H) Macrophages M2. (I–L) Functional stratification based on *KLRB1* expression. Boxplots display differences in (I) Follicular helper T cells (elevated in *KLRB1*-high for both diseases), (J) Activated NK cells, (K) Resting NK cells, and (L) Activated memory CD4 T cells. Statistical significance between expression strata was assessed using the Wilcoxon rank-sum test (∗*P* < 0.05, ∗∗*P* < 0.01, ∗∗∗*P* < 0.001, ∗∗∗∗*P* < 0.001, ns: not significant). **Abbreviations:** SLE, Systemic Lupus Erythematosus; IPF, Idiopathic Pulmonary Fibrosis; NK, Natural Killer.

Immune cell fractions were inferred by CIBERSORT in the SLE (GSE49454) and IPF (GSE33566) cohorts. Sample-level composition profiles showed marked inter-individual heterogeneity in both diseases (Fig. 7A). Case–control comparisons across the 22 leukocyte subsets identified multiple differentially represented cell types within each cohort (Fig. 7C), and effect sizes quantified by Cliff’s delta highlighted cross-disease concordance. Specifically, Monocytes and activated NK cells were consistently increased in cases, whereas naïve B cells, naïve CD4 T cells, and resting NK cells were consistently decreased, forming a shared immune signature across SLE and IPF (Fig. 7D). In parallel, Spearman correlation analysis between candidate genes and immune fractions revealed structured gene–cell association patterns in both cohorts (Fig. 7B), supporting coordinated links between the identified hub genes and immune infiltration phenotypes.

To functionally connect hub-gene expression to immune-cell shifts, we performed within-cohort median-split stratification among case samples. In the GRN stratification (myeloid-focused validation), the GRN-high group displayed higher neutrophil fractions in both cohorts (Fig. 7E). Additional myeloid signals showed cohort specificity: in IPF, macrophages M0 and monocytes were enriched in the GRN-high group (Fig. 7F and G), whereas in SLE, macrophages M2 were increased in the GRN-high group (Fig. 7H). In the KLRB1 stratification (lymphoid/NK-focused validation), T follicular helper cells were consistently enriched in the KLRB1-high group in both cohorts (Fig. 7I). Moreover, resting NK cells were higher in the KLRB1-high group in both cohorts (Fig. 7K), while in SLE, activated NK cells were reduced and activated CD4 memory T cells were increased in the KLRB1-high group (Fig. 7J and L). Together, these results link GRN to a myeloid/neutrophil-skewed inflammatory state and connect KLRB1 to lymphoid/NK–T cell compartment remodeling, providing an immune-cell layer of evidence consistent with the functional signals identified upstream.

### 3.8 Clinical validation of the GRN-centered signature and external specificity assessment

Baseline characteristics of the hospital validation cohort are summarized in Table 2. The cohort comprised 60 patients with SLE and 30 age-/sex-matched healthy controls (HCs), with comparable age, sex distribution, and BMI. As expected, SLE patients showed markedly lower WBC counts and complement levels and higher inflammatory indices, including ESR and hs-CRP (all *P* < 0.001). Serum GRN concentrations were also substantially higher in SLE than in HCs (median [IQR]: 43.58 [38.44–54.42] vs. 14.26 [12.79–15.26] ng/mL; *P* < 0.001; Table 2). Most SLE patients were receiving contemporary therapy at sampling, including glucocorticoids (98.3%) and hydroxychloroquine (86.7%), with additional immunosuppressant/biologic exposure in a subset (Table 2).

**Table 2:** Clinical characteristics of the hospital validation cohort.

Characteristics	Healthy Controls	SLE Patients	*P*-value
**Demographics**			
Age (years)	39.50 (27.00, 48.00)	43.00 (28.00, 49.00)	0.540
Female	28 (93.3%)	57 (95.0%)	1.000
BMI (kg/m^2^)	22.34 ± 2.41	23.73 ± 4.52	0.061
**Lifestyle and recent exposures**			
Current smoker	4 (13.3%)	6 (10.0%)	0.726
Recent infection (≤2 weeks)	2 (6.7%)	8 (13.3%)	0.486
Recent antibiotics (≤2 weeks)	2 (6.7%)	7 (11.7%)	0.712
**Laboratory measurements**			
WBC ( × 10ˆ9/L)	6.59 ± 1.35	2.77 ± 1.01	<0.001
ESR (mm/h)	8.97 ± 3.28	29.65 ± 9.54	<0.001
hs-CRP (mg/L)	0.86 (0.74, 0.96)	3.97 (2.35, 7.56)	<0.001
C3 (g/L)	1.22 (1.10, 1.37)	0.40 (0.26, 0.57)	<0.001
C4 (g/L)	0.24 (0.20, 0.30)	0.13 (0.08, 0.18)	<0.001
Serum GRN (ELISA)	14.26 (12.79, 15.26)	43.58 (38.44, 54.42)	<0.001
**Disease characteristics (SLE only)**			
Disease duration (years)	—	10.60 (6.57, 21.50)	—
SLEDAI	—	7.30 (6.17, 8.55)	—
Lupus nephritis	—	12 (20.0%)	—
Arthritis	—	26 (43.3%)	—
Skin/mucosal involvement	—	47 (78.3%)	—
Hematologic involvement	—	31 (51.7%)	—
Anti-dsDNA positive (≥1)	—	56 (93.3%)	—
Anti-dsDNA: Negative	—	4 (6.7%)	—
Anti-dsDNA: Weak positive	—	6 (10.0%)	—
Anti-dsDNA: Positive	—	43 (71.7%)	—
Anti-dsDNA: Strong positive	—	7 (11.7%)	—
**Current medications**			
Glucocorticoids (current)	0 (0.0%)	59 (98.3%)	<0.001
prednisone	—	33 (55.0%)	—
methylprednisolone	—	26 (43.3%)	—
NA	—	1 (1.7%)	—
GC dose (mg/day, as prescribed)	—	16.00 (11.50, 21.25)	—
Prednisone-equivalent dose (mg/day)	—	18.75 (14.38, 25.00)	—
Hydroxychloroquine (current)	0 (0.0%)	52 (86.7%)	<0.001
Mycophenolate mofetil (MMF, current)	0 (0.0%)	21 (35.0%)	<0.001
Azathioprine (AZA, current)	0 (0.0%)	4 (6.7%)	0.297
Cyclophosphamide (CYC, current)	0 (0.0%)	0 (0.0%)	1.000
Biologics (current)	0 (0.0%)	3 (5.0%)	0.548
Recent IV pulse (≤4 weeks)	0 (0.0%)	0 (0.0%)	1.000

HC (Group = 0): 30 participants; SLE (Group = 1): 60 participants.

Notes:

Continuous variables are presented as mean ± SD if approximately normally distributed; otherwise as median (Q1, Q3). Categorical variables are presented as n (%).

P-values are two-sided and were not adjusted for multiple comparisons. For between-group comparisons, Student’s t-test or Wilcoxon rank-sum test was used for continuous variables, and χ^2^ test or Fisher’s exact test was used for categorical variables, as appropriate.

Variables only applicable to SLE were summarized descriptively in the SLE group and not tested against HC.

**Fig. 8:**
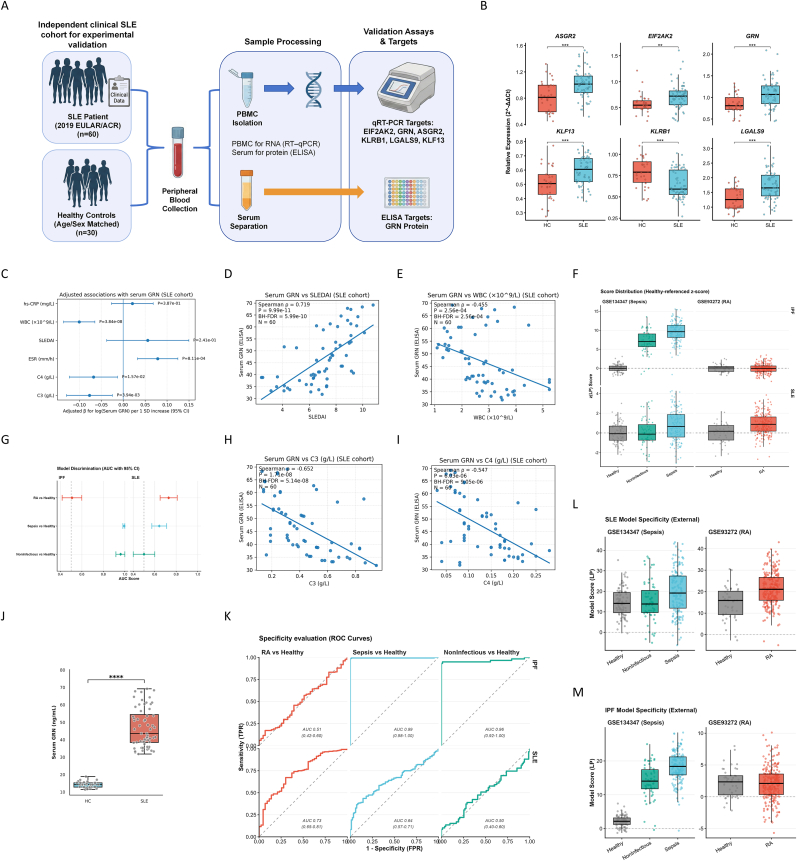
**Clinical validation of the GRN-centered signature and external specificity assessment in inflammatory cohorts.** **(A)** Schematic overview of the independent clinical validation study design. Peripheral blood samples were collected from 60 SLE patients and 30 age/sex-matched healthy controls. PBMCs were isolated for gene expression analysis (RT-qPCR), and serum was separated for protein quantification (ELISA). **(B)** Forest plot summarizing the covariate-adjusted associations between serum GRN levels and key clinical indices in the SLE cohort. Multivariable linear regression models were adjusted for age, sex, medication exposure (prednisone/hydroxychloroquine/immunosuppressants), and inflammatory confounders. Points represent standardized *β* coefficients (change in log(Serum GRN) per 1 SD increase in the index), and error bars indicate 95% confidence intervals. **(C)** Scatter plots illustrating the correlations between serum GRN levels and clinical parameters in SLE patients: SLEDAI score, ESR (mm/h), hs-CRP (mg/L), C3 (g/L), C4 (g/L), and WBC count (×10^9^/L). Spearman’s rank correlation coefficients (*ρ*) and Benjamini–Hochberg adjusted *P*-values (BH-FDR) are shown. **(D)** Boxplots showing the relative expression levels of the six signature genes (*ASGR2, EIF2AK2, GRN, KLF13, KLRB1, LGALS9)* in PBMCs from healthy controls (HC) and SLE patients, quantified by RT-qPCR. Expression was normalized to *β*-actin (2^-△△*Ct*^). **(E)** Specificity evaluation of the diagnostic models in external non-target inflammatory cohorts (GSE93272: Rheumatoid Arthritis [RA]; GSE134347: Sepsis and Non-infectious Critical Illness). **Top panel (ROC curves):** Receiver Operating Characteristic curves assessing the discrimination of the IPF model (top row) and SLE model (bottom row) in differentiating RA, Sepsis, or Non-infectious illness from healthy controls. **Bottom panel (AUC Forest Plot):** Forest plot displaying the Area Under the Curve (AUC) with 95% confidence intervals for each comparison. **(F)** Boxplots displaying the distribution of model scores (Healthy-referenced z-scores of the linear predictor) in the external inflammatory cohorts. **Top panel:** IPF model scores. **Bottom panel:** SLE model scores. The dashed line at y = 0 represents the healthy reference mean. **Abbreviations:** SLE, Systemic Lupus Erythematosus; PBMC, Peripheral Blood Mononuclear Cell; GRN, Progranulin; SLEDAI, SLE Disease Activity Index; ESR, Erythrocyte Sedimentation Rate; hs-CRP, high-sensitivity C-reactive Protein; WBC, White Blood Cell; ROC, Receiver Operating Characteristic; AUC, Area Under the Curve.

In PBMCs, RT–qPCR validated dysregulation of the transcriptome-derived six-gene panel. ASGR2, EIF2AK2, GRN, KLF13, and LGALS9 were significantly upregulated in SLE, whereas KLRB1 was downregulated (all Wilcoxon *P* < 0.01; Fig. 8B). Consistently, ELISA confirmed a pronounced elevation of circulating GRN in SLE compared with HCs (Fig. 8J).

Within SLE patients, serum GRN exhibited strong associations with disease activity and serological indices. Spearman analyses showed a positive correlation with SLEDAI (ρ = 0.719, BH–FDR = 5.99 × 10^−10^; Fig. 8D), and inverse correlations with complement C3 (*ρ* = −0.652, BH–FDR = 5.14 × 10^−8^; Fig. 8H) and C4 (*ρ* = −0.547, BH–FDR = 9.05 × 10^−6^; Fig. 8I). Serum GRN also correlated negatively with WBC counts (*ρ* = −0.455, BH–FDR = 2.56 × 10^−4^; Fig. 8E). In bivariate analyses, serum GRN showed a positive correlation with ESR (*ρ* = 0.566, *P* = 2.45 × 10^−6^; BH–FDR = 4.90 × 10^−6^; n = 60) and hs-CRP (*ρ* = 0.489, *P* = 7.40 × 10^−5^; BH–FDR = 8.88 × 10^−5^; n = 60) (Supplementary Figs. S1–S2). To quantify covariate-adjusted associations, multivariable linear models were fitted with log-transformed serum GRN as the outcome and one Z-standardized clinical index entered per model. After adjustment for demographics, contemporaneous medication exposure, and inflammatory/infectious confounding as specified in Methods, serum GRN remained independently associated with WBC (*P* = 3.84 × 10^−8^), ESR (*P* = 8.11 × 10^−4^), C3 (*P* = 3.94 × 10^−3^), and C4 (*P* = 1.57 × 10^−2^), whereas associations with hs-CRP (*P* = 0.387) and SLEDAI (*P* = 0.241) were attenuated (Fig. 8E; covariate-adjusted models).

To characterize model behavior in external inflammatory contexts, the fixed SLE and IPF classifiers were applied without refitting to an RA whole-blood cohort (GSE93272) and an ICU cohort including sepsis and non-infectious critical illness (GSE134347) (Table S2). The SLE model showed moderate discrimination for RA versus healthy controls (AUC 0.73, 95% CI 0.65–0.81) but limited discrimination for sepsis versus healthy controls (AUC 0.64, 95% CI 0.57–0.71) and no discrimination for non-infectious critical illness versus healthy controls (AUC 0.50, 95% CI 0.40–0.60) (Fig. 8K), consistent with the external score distributions (Fig. 8F–L). In contrast, the IPF model showed minimal discrimination for RA versus healthy controls (AUC 0.51, 95% CI 0.42–0.60) but strong discrimination for ICU sepsis (AUC 0.99, 95% CI 0.98–1.00) and ICU non-infectious critical illness (AUC 0.96, 95% CI 0.92–1.00) versus healthy controls (Fig. 8K), mirrored by marked score elevations in ICU groups (Fig. 8F–M).

## 4 Discussion

In this study, we used an integrative framework to compare peripheral-blood transcriptomic programs between SLE and IPF. We combined cross-disease differential expression, WGCNA-based co-expression analysis, and consensus feature selection to prioritize direction-consistent shared signals and to derive compact diagnostic panels for each disease. The resulting six-gene SLE panel and four-gene IPF panel showed strong discrimination in the discovery cohorts and retained performance in multiple independent public datasets. In an independent clinical SLE cohort, PBMC assays supported the dysregulation of key signature genes, including an inverse pattern of GRN upregulation and KLRB1 downregulation. Serum progranulin was also elevated in SLE and tracked with clinical and serological indices, with several associations persisting after covariate adjustment. Collectively, our results point to a shared peripheral-blood state characterized by heightened myeloid-associated activity alongside reduced CD161-associated lymphoid signals across the SLE–IPF spectrum.

GRN was one of the most consistent directionally upregulated signals shared by SLE and IPF in our analysis. GRN encodes progranulin, a secreted protein with established roles in lysosome-linked immune regulation and tissue repair. In human critical illness, progranulin biology is strongly engaged [30]. A comparative observational study reported distinct progranulin-associated patterns across sepsis, community-acquired bacterial pneumonia, and COVID-19, supporting its sensitivity to high-intensity innate immune activation states [31]. This context helps interpret our specificity experiments. When we applied fixed models to non-target ICU cohorts, the IPF panel showed very high discrimination, which is compatible with the idea that GRN can track severe myeloid-driven inflammation that is not disease-exclusive. At the same time, experimental work in fibrotic lung injury also links progranulin to inflammatory and profibrotic signaling, including IL-6 and TGF-β pathway activity, providing a plausible route for its upregulation in fibrosing lung pathology [32]. Collectively, these lines of evidence suggest that GRN is a biologically credible “severity-responsive” myeloid node across the SLE–IPF spectrum, but it also highlights why infection and critical illness must be explicitly considered when interpreting GRN-centered blood signatures.

In contrast to the shared upregulation of GRN, we observed a directionally consistent reduction of KLRB1 across SLE and IPF blood transcriptomes. KLRB1 encodes CD161 (NKR-P1A), a C-type lectin-like receptor enriched in NK cells and MAIT-lineage T cells and linked to cytotoxic and tissue-surveillance programs [33]. Because KLRB1 is strongly cell-type associated, lower bulk expression is most plausibly explained by shifts in the abundance or distribution of CD161-positive lymphocyte compartments rather than a uniform downshift within all leukocytes [34].

This interpretation is compatible with immunophenotyping literature summarized in MAIT-focused autoimmune reviews, where CD161-high T-cell subsets are frequently perturbed in systemic autoimmunity, including SLE [35]. In parallel, peripheral-blood single-cell profiling in progressive IPF has reported broad immune remodeling with relative depletion of lymphocyte populations and enrichment of myeloid programs, a context in which reduced CD161-associated signals are biologically expected [36]. Together with GRN upregulation, the GRN-high/KLRB1-low pattern therefore supports a composite circulating state characterized by enhanced innate activation with attenuated lymphoid surveillance, which may also help explain why model behavior changes when applied to ICU inflammatory states [37]. Notably, CD161 is a membrane receptor and is not routinely assayed as a soluble serum protein; thus, our study prioritized transcript-level evidence, while targeted flow cytometry of CD161-positive NK/MAIT subsets would be an appropriate next step for mechanism-aligned validation.

To maximize robustness, we used a consensus feature-selection strategy that integrates LASSO, SVM–RFE, and random forest to derive parsimonious panels, and we evaluated performance using external cohorts rather than relying on discovery estimates alone. As expected for transcriptome-based classifiers, discrimination decreased in independent datasets, which is commonly driven by platform effects (e.g., microarray versus RNA-seq), cohort case-mix, and sampling context heterogeneity [38]. Importantly, the non-target analyses were not intended as “diagnostic substitutions”, but as stress tests of specificity under realistic inflammatory confounding. The SLE panel showed moderate separation in rheumatoid arthritis yet little separation in non-infectious critical illness, consistent with a shared autoimmune-adaptive blood program across systemic rheumatic diseases rather than a generic pan-inflammation signal [39]. In contrast, the IPF panel displayed minimal separation in RA but very strong separation in ICU cohorts, a pattern compatible with a myeloid-dominant severity axis and with the GRN-centered biology discussed above [40,41]. Taken together, these results define a practical boundary for interpretation: the panels should be read as context-dependent immune-state scores, and their clinical meaning depends on the comparator group and on whether infection or critical illness is present.

Our hospital cohort adds a direct clinical layer to the transcriptome-derived findings. Serum progranulin was markedly increased in SLE, and PBMC assays supported concordant GRN upregulation, which strengthens the biological credibility of the blood signature beyond public datasets [42]. Within SLE, serum progranulin aligned with immunologic activity, including inverse associations with complement levels (C3/C4) and relationships with inflammatory and hematologic indices. Importantly, these links with complement consumption remained detectable after covariate adjustment that accounted for key clinical confounders such as glucocorticoid exposure and concomitant infection. In contrast, associations with acute-phase reactants were weaker after adjustment, suggesting that progranulin captures a myeloid-linked immune activation state that is related to, but not fully explained by, non-specific systemic inflammation [43]. From a translational perspective, GRN therefore appears more suitable as an adjunct readout of immune-state remodeling than as a standalone disease marker. In practice, it could complement routine serology when clinical activity and conventional markers are discordant, and it may help identify patients enriched for sustained innate activation that is also implicated in tissue injury and remodeling pathways [20]. However, our validation is cross-sectional and does not test longitudinal outcomes. Any inference about lung-fibrosis risk in SLE would require prospective SLE-ILD–phenotyped cohorts, standardized infection adjudication, and ideally paired tissue or single-cell profiling to link circulating signals to lung pathology [44].

Several limitations should be considered when interpreting these findings. First, we utilized IPF as a pragmatic reference for fibrosing lung biology; however, it is not a perfect surrogate for SLE-associated ILD. Therefore, our cross-disease convergence should be viewed as a hypothesis-generating map of shared circulating programs rather than direct validation of an SLE-ILD biomarker. Second, the attenuation of model performance in external cohorts reflects the inherent heterogeneity of public retrospective datasets, where platform effects (microarray vs. RNA-seq), variable sampling protocols, and unadjusted medication exposures introduce residual confounding difficult to fully eliminate. Third, the downregulation of KLRB1 in bulk transcriptomes carries inherent ambiguity, as it may reflect shifts in the abundance of CD161-positive NK/MAIT compartments, altered activation states, or both. Resolving the cellular origin of the GRN-high/KLRB1-low pattern requires targeted flow cytometry and single-cell profiling with standardized gating. Fourth, our hospital validation was cross-sectional. While it supports biological plausibility, it does not establish temporal precedence or prognostic value. Prospective SLE cohorts with standardized HRCT surveillance and pulmonary function follow-up are needed to determine whether this signature identifies patients at risk of progressive lung remodeling. Finally, future work expanding specificity testing to other connective tissue disease–associated ILDs would help refine the clinical boundary of these immune-state scores, clarifying their incremental value beyond existing serology and imaging.

## 5 Conclusions

In conclusion, this study defines a reproducible peripheral-blood transcriptomic signature shared by SLE and IPF, with progranulin (GRN) and CD161 (KLRB1) emerging as convergent anchors. The GRN-high/KLRB1-low pattern is consistent with a composite circulating immune state marked by enhanced myeloid-associated activity together with reduced CD161-associated lymphoid signals. Using consensus feature selection and external evaluation across independent cohorts, we derived parsimonious panels that generalize beyond the discovery datasets and show context-dependent specificity. Clinically, serum progranulin aligned with immunologic activity in SLE, including complement consumption, with key associations persisting after adjustment for major clinical confounders. Together, these findings provide a testable framework for cross-disease immune-state stratification and motivate prospective, phenotype-defined studies to determine longitudinal utility across the SLE–IPF spectrum.

## CRediT authorship contribution statement

**Lijun Pang:** Writing – review & editing, Writing – original draft, Visualization, Software, Investigation, Formal analysis, Data curation, Conceptualization. **Yunfei Li:** Writing – original draft, Methodology, Data curation, Conceptualization. **Junjie Chen:** Writing – original draft, Visualization, Validation, Software, Data curation. **Shuangshuang Shang:** Writing – original draft, Validation, Supervision, Software. **Ming Li:** Writing – original draft, Supervision, Software, Funding acquisition, Data curation. **Chuanbing Huang:** Writing – review & editing, Writing – original draft, Supervision, Funding acquisition, Conceptualization.

## Ethics approval and consent to participate

This study was approved by the Ethics Committee of The First Affiliated Hospital of Anhui University of Chinese Medicine (Approval No. 2024AH-08). Participants were prospectively recruited between March 2025 and September 2025, and written informed consent was obtained from all participants prior to enrollment. The study was conducted in accordance with the Declaration of Helsinki.

## Declaration of generative AI and AI-assisted technologies in the writing process

During the preparation of this work the authors used ChatGPT (OpenAI) in order to improve language and readability and assist with manuscript drafting/editing. After using this tool, the authors reviewed and edited the content as needed and take full responsibility for the content of the published article.

## Funding

This work was supported by the 10.13039/501100001809National Natural Science Foundation of China (grant No. 82574970); the Anhui Provincial Clinical Medical Research and Translational Program (grant No. 202304295107020115); the Special Project of the Institute of Xin’an Medicine and Modernization of Traditional Chinese Medicine, Institute of Big Health, 10.13039/501100009004Anhui University of Chinese Medicine (grant No. 2023CXMMTCM015); the 2024 Key Scientific Research Project of Higher Education Institutions in Anhui Province (grant No. 2024AH050957); and the 2024 Scientific Research Project of the Anhui Provincial Health Commission (grant No. 2024Aa30428).

## Declaration of competing interest

The authors declare that they have no known competing financial interests or personal relationships that could have appeared to influence the work reported in this paper.

## Figures and Tables

**figs1:**
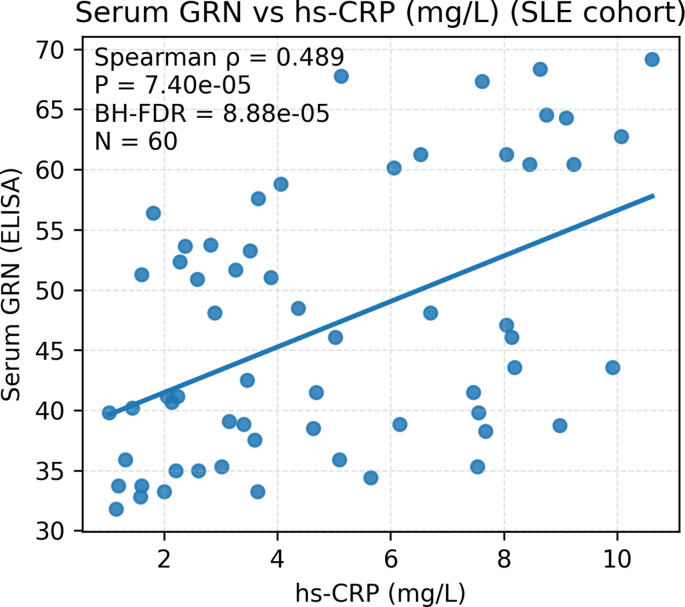


**figs2:**
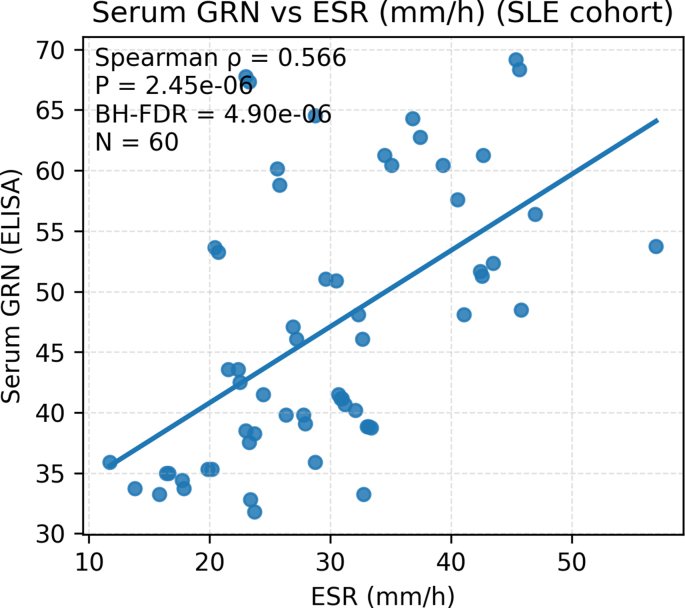


## Data Availability

Data will be made available on request.
