# Supplementary material for: GRN and KLRB1 define a shared peripheral-blood transcriptomic signature linking SLE and IPF
Source: J Transl Autoimmun. 2026 Feb 20;12:100357. doi: 10.1016/j.jtauto.2026.100357 (PMC12955197; doi:10.1016/j.jtauto.2026.100357)
Supplement: Multimedia component 1 [file mmc1.docx]

Table S1: Primer for each detection index

| Gene | Amplicon Size  （bp） | Forward primer  （5'→3'） | Reverse primer  （5'→3'） |
| --- | --- | --- | --- |
| β-actin | 96 | CCCTGGAGAAGAGCTACGAG | GGAAGGAAGGCTGGAAGAGT |
| GRN | 104 | GACAAATGGCCCACAACAC | GAAGTCCCTGAGACGGTAAAG |
| LGALS9 | 148 | TTGTTAAGTCGTTCCCTC | ACCTCCTTGAATAGTCCC |
| KLF13 | 197 | GCACAAGTGCCACTACGC | GCAGATGTCCAGGAAAGAG |
| ASGR2 | 119 | TCCCAGGCCAGGAGGTGCCAGGTTA | TAACCTGGCACCTCCTGGCCTGGGA |
| KLRB1 | 145 | TCCACCAAAGAATCCAGC | AGCCGTTTATCCACTTCC |
| EIF2AK2, | 167 | GCTTTGGAAATGTCTGACGGT | GCTTGCTGTTGATAGAACACTG |
